# Benefits and challenges of the integration of haptics‐enhanced virtual reality training within dental curricula

**DOI:** 10.1002/jdd.13800

**Published:** 2024-12-17

**Authors:** Szabolcs Felszeghy, Murat Mutluay, Mikko Liukkonen, Nicla Flacco, Mahmoud M. Bakr, Sarah Rampf, Simona‐Georgiana Schick, Faisal Mushtaq, Maria F. Sittoni‐Pino, Kristin Ackerman, Santiago Arias‐Herrera, Ben Audsley, Kinga Bágyi, Santiya Bell, Tamás Bistey, Samantha Byrne, Giorgia Carpegna, Esther Carramolino‐Cuéllar, Juliana B. da Costa, Mark R. Durham, Sónnica Galán‐Gil, Gábor Gerber, Diego González‐Carrasco, Kandace Gourley, Péter Hermann, Outi Huhtela, Hanna Hytönen, Antti Kämppi, Michael Lampe, Carlos López‐Roig, Rita Marincsák, David Morton, Masako Nagasawa, Katalin Nagy, László Nagy, Marit Øilo, Cesar Orsini, Ulla Palotie, Mihaela Pantea, Damiano Pasqualini, Anita Pétercsák, Daniela Pino‐Valenzuela, Edgar Quenta‐Silva, Amitha Ranauta, Gitana Rederiene, Pere Riutord‐Sbert, Ewa J. Rodakowska, María P. Rodríguez‐Hopp, Mauricio Saenz‐Laguna‐Saavedra, Anna L. Suominen, Jorge Tricio, Ülle Voog‐Oras, Michael D. Wolcott, Sila Nur Usta, Peter Lingström, Muhammad A. Shazib, Maria C. Manzanares‐Céspedes, Thomas J. Greany, Margrit Maggio, Rebecca Stolberg, Gül Gülsün, Sompop Bencharit, Barry Quinn

**Affiliations:** ^1^ Institute of Dentistry, School of Medicine University of Eastern Finland Kuopio Finland; ^2^ Institute of Clinical Medicine, School of Medicine University of Eastern Finland Kuopio Finland; ^3^ Faculty of Health Sciences Universidad Europea de Valencia Valencia Spain; ^4^ School of Medicine and Dentistry Griffith University Gold Coast Queensland Australia; ^5^ Department of Conservative Dentistry, Clinic for Oral, Dental and Maxillofacial Diseases, Heidelberg Medical Faculty Heidelberg University Heidelberg Germany; ^6^ Centre for Immersive Technologies University of Leeds & NIHR Leeds Biomedical Research Centre Leeds UK; ^7^ NIHR Biomedical Research Centre Leeds UK; ^8^ Workman School of Dental Medicine High Point University High Point North Carolina USA; ^9^ Queen Marry University of London London UK; ^10^ Department of Operative Dentistry and Endodontics, Faculty of Dentistry University of Debrecen Debrecen Hungary; ^11^ Department of Prosthodontics, Faculty of Dentistry University of Debrecen Debrecen Hungary; ^12^ Melbourne Dental School University of Melbourne Melbourne Victoria Australia; ^13^ Department of Surgical Sciences University of Turin, Dental School Turin Italy; ^14^ Department of Oral Rehabilitation and Biosciences, School of Dentistry Oregon Health & Science University Portland Oregon USA; ^15^ School of Dentistry University of Utah Salt Lake City Utah USA; ^16^ 2nd Department of Anatomy Semmelweis University Budapest Hungary; ^17^ ADEMA‐HEALTH Group IUNICS University of the Balearic Islands Palma Spain; ^18^ Clinic for Prosthodontics, Faculty of Dentistry Semmelweis University Budapest Hungary; ^19^ Department of Oral and Maxillofacial Diseases University of Helsinki and Helsinki University Hospital Helsinki Finland; ^20^ School of Dental Medicine University of Colorado Anschutz Medical Campus Aurora Colorado USA; ^21^ University of Utah School of Medicine Salt Lake City Utah USA; ^22^ Division of Bio‐Prosthodontics Faculty of Dentistry & Graduate School of Medical and Dental Sciences Niigata University Niigata Japan; ^23^ School of Dentistry University of Szeged Szeged Hungary; ^24^ Department of Clinical Dentistry University of Bergen Bergen Norway; ^25^ Centre for Dental Development and Research, Norwich Medical School University of East Anglia Norwich UK; ^26^ Faculty of Dentistry Universidad de los Andes Santiago Chile; ^27^ Department of Fixed Prosthodontics and Occlusology, Faculty of Dentistry “Carol Davila” University of Medicine and Pharmacy Bucharest Romania; ^28^ Department of Surgical Sciences University of Turin Turin Italy; ^29^ Department of Operative Dentistry, Faculty of Dentistry University of Concepción Concepción Chile; ^30^ Facultad de Estomatología Universidad Peruana Cayetano Heredia Lima Peru; ^31^ European Dental Hygienists Federation Utrecht Netherlands; ^32^ Facultad de Salud y Odontología Universidad Diego Portales Santiago Chile; ^33^ Oral Health Teaching Unit Kuopio University Hospital Kuopio Finland; ^34^ Faculty of Health and Dentistry Universidad de los Andes Santiago Chile; ^35^ Institute of Dentistry University of Tartu Tartu Estonia; ^36^ Gulhane Faculty of Dentistry University of Health Sciences Ankara Ankara Turkey; ^37^ Department of Cariology, Institute of Odontology, Sahlgrenska Academy University of Gothenburg Gothenburg Sweden; ^38^ Human Anatomy and Embryology Unit, Faculty of Medicine and Health Sciences University of Barcelona Barcelona Spain; ^39^ Department of Restorative Dentistry, School of Dental Medicine University of Colorado Anschutz Medical Campus Aurora Colorado USA; ^40^ Division of Restorative Dentistry, School of Dental Medicine University of Pennsylvania Philadelphia Pennsylvania USA; ^41^ American Dental Education Association Washington District of Columbia USA; ^42^ Faculty of Health & Life Sciences, Institute of Life Course and Medical Sciences, School of Dentistry University of Liverpool Liverpool UK

**Keywords:** evidence‐based practice, haptics‐reinforced immersive learning, manual dexterity assessment, simulation training

## Abstract

**Background:**

Haptics‐enhanced virtual reality (VR‐haptic) simulation in dental education has evolved considerably during the past decade, representing a promising resource of simulation‐based training opportunities to support conventional practice. We aim to summarize current literature on the applications of VR‐haptics in learning, practicing, and teaching dental education.

**Methods:**

A literature search was performed using PubMed, focusing on research articles published between January 2010 and January 2024. Out of the 667 articles that matched the search terms (dentistry, education, haptic, teaching, training, virtual reality), 105 were screened, and 42 were eligible for full‐text reading and utilization. Findings from an ongoing educator survey on the use of VR‐haptics in dental education have also been provided.

**Results:**

VR‐haptic simulation has been shown to have a supportive role in dental simulation practice. Despite training within a digital world, hand skill transfer to the real world has been demonstrated, which affords educators more flexibility in how to train their students before and during traditional preclinical and clinical practical education. The individualized VR‐haptic training and feedback help students in mastering essential working techniques, while also increasing engagement and motivation.

**Conclusions:**

VR‐haptics‐supported dental education can help students effectively address challenges during their preclinical and clinical training, as well as in their subsequent careers, and it may help mitigate some weaknesses of the current educational system. Validation is a key factor for the acceptance of VR‐haptic simulators; thus, further research and verification are needed before VR‐haptics could be considered a primary hand skill development method of learning in dental education. VR‐haptic simulation may in the future be used as an assessment tool for the students’ and clinicians’ credentialing process.

## 1 INTRODUCTION

Dentistry is a highly complex profession with long learning curves necessary for achieving optimal outcomes.^1^ It has over 9000 years of history, with excavated remains from the Indus valley yielding evidence of dentistry being practiced in the Neolithic age.^2^ The earliest evidence of a dental restoration, made of beeswax, was discovered in Slovenia, and has been dated to be from 5800 years ago.^3^ Although ancient, this scientific discipline is highly adaptive and has undergone numerous changes, which have presented significant challenges in both training and measuring fine motor skill proficiency of students who are learning the dental profession.

Preclinical dental training exercises, whether they be in the form of observation or practice, have mainly been taught using real or synthetic teeth set placed in a phantom head since this methodology was introduced in 1894 by Oswald Fergus.^4^ This highly practice‐oriented, conventional didactic methodology has been widely utilized and emphasizes the central role of dental educators in the transferring of knowledge and practices with large amounts of theoretical content in a simulation environment.^5^, ^6^ However, given the advances in the past decades in digital technologies, and the adoption of student‐centered curriculum design, this traditional landscape has slowly shifted to modern digital simulation. The first haptics‐reinforced virtual reality‐based dental trainer, Simodont, was developed through cooperation between the Academic Centre for Dentistry Amsterdam (ACTA) and Fokker Control Systems, later Moog Inc., and now owned by Nissin Dental Products Inc., with the first finalized unit installed at ACTA in 2009.^7^, ^8^ Today, the unquestionable shift toward the adoption and integration of haptics‐enhanced virtual reality (VR‐haptic) digital technologies is evident.^9^

While this article will continue to use the term VR‐haptics for the sake of internal consistency, these simulators belong under the umbrella of extended reality (XR), which is a comprehensive term that encompasses a variety of immersive technologies aimed at enhancing or altering our perception of reality through the integration of digital elements.^10^, ^11^ XR can be classified into three primary categories: augmented reality (AR), mixed reality (MR), and virtual reality (VR), in order of how much of what one sees only exists digitally. AR equipment overlays digital elements over physical objects, MR devices enable these elements to interact, and in VR all interactions happen in a digital space. Each of these technologies provides unique experiences and applications, significantly impacting a diverse array of industries like education, entertainment, healthcare, and training. Haptics, on the other hand, is a multidisciplinary field dedicated to the science and technology of transmitting and interpreting information through touch. This involves generating a variety of tactile sensations, typically either electronically or mechanically, to enable users to receive feedback while interacting with different devices and interfaces.^12^ The term “haptics” originates from the Greek word “haptesthai,” meaning “to touch,” emphasizing its fundamental link to tactile perception. Different types of VR‐haptic trainers are now available in dental education training. Several major companies are competing in the development of commercial VR‐haptic simulators, including Acadental, Nissin Dental Products, SIMtoCARE, UNI SIM, and Virteasy Dental.

The benefits of VR and haptics‐reinforced dental health education appear to be far‐reaching, providing much‐needed assistance to students, clinicians, patients, and educators alike.^13^, ^14^, ^15^, ^16^ VR‐haptic simulation helps bridge the gap between theoretical visualization, preclinical, and clinical hands‐on training. Thus, it has the potential to overcome some of the shortcomings of the current master–apprentice training model that has been used as a standard in dental education. VR‐haptic simulation emphasizes active learning and hands‐on learning to develop skills necessary for clinical procedures. Thus, it allows educators to guide students in performing dental exercises within a standardized, VR‐enhanced, safe, and risk‐free digital environment.^17^, ^18^, ^19^, ^20^ As material losses are no longer a factor while the students practice their fine motor skills, VR‐haptic simulation reduces students’ anxiety due to fear of failure. With the help of VR‐haptic training, individualized learning is possible as teachers can freely focus on the students who are in greater need of help. At the same time, fast learners can practice a higher level skill or focus on other skills. Students can independently practice on their own time and pace, outside of teaching hours, as the simulators can provide real‐time, objective feedback based on the students’ performances. In this context, the utilization of haptic simulators to differentiate between students’ proficiency levels and for performance prediction has the potential to facilitate personalized training.^15^ The VR‐haptic environment allows for the standardization of student experiences, and provides opportunities for objective and consistent assessment. The benefits of VR‐haptic practice lie in its relative casualness, the ability to try again as many times as necessary, and the possibility of training at a time suitable for each student. Haptics‐based simulators offer visuo‐haptic output to evoke sensorial experiences that allow the user to receive tactile feedback based on the different properties of the simulated objects, providing dentistry students experiences that are akin to what they might encounter during real patient care. For specific clinical procedures, VR‐haptic simulation can provide the opportunity for realistic training of clinical situations, as unique patient cases could be scanned and uploaded into the system for students to train on prior to a real clinical encounter.

VR‐haptic technology offers significant advantages in advancing dental education by facilitating enhanced skill acquisition and boosting student confidence as discussed above. However, challenges related to financial investment, integration into existing curricula, and skepticism among educators remain barriers to its broader implementation.^21^ A comparison of the current advantages and challenges is summarized in Table 1. Opponents of VR‐haptic technology in dental education argue that it may lack the realism and nuanced tactile feedback of hands‐on practice with actual dental materials and patients, potentially leading to an over‐reliance on virtual experiences. It is believed that true mastery of fine motor skills in dentistry requires extensive real‐world practice that simulation may not adequately replicate, especially for complex or unpredictable clinical scenarios. Furthermore, they contend that students may become overly accustomed to the risk‐free nature of virtual environments, which could lead to hesitation or mistakes in real patient interactions. There are also concerns about accessibility and cost, as VR‐haptic systems can be expensive to implement and maintain, potentially limiting access for institutions and students in lower resourced settings. While noteworthy and important to keep in mind, these critiques may become less relevant as VR‐haptic technology advances, refining tactile accuracy and cost‐effectiveness. In fact, as research increasingly supports the potential of VR‐haptics to complement traditional methods, providing safe, flexible, and objective skill‐building environments, VR‐haptics may ultimately be seen as an essential, rather than supplementary, component of dental education.

**TABLE 1:** A brief comparison of the current advantages and disadvantages of VR‐haptics.

Positive outcomes	Opposing views
**Enhanced skill development**: VR‐haptic simulators provide realistic tactile feedback, allowing students to practice dental procedures in a controlled environment. This feedback helps develop essential motor skills and hand–eye coordination, which are crucial for performing real‐life dental tasks effectively. (Patil et al., 2023) **Increased confidence**: Many studies indicate that students who utilize VR‐haptic technology report increased self‐confidence in their abilities. The immersive training experience allows them to practice repeatedly without the risk of harming real patients, thereby enhancing their readiness for clinical practice. (Felszeghy et al., 2023) **Improved learning outcomes**: Research shows that combining traditional training methods with VR‐haptic technology can lead to better learning outcomes. For instance, a study at the University of Eastern Finland found that students who practiced with VR haptics alongside conventional methods demonstrated improved performance in tooth preparation tasks. (Hsu and Chang, 2023) **Real‐time feedback**: Haptic simulators can provide instant feedback on various parameters such as pressure and technique, allowing students to refine their skills and understand the nuances of different dental procedures. (Felszeghy et al., 2023) **Comprehensive training environment**: Advanced haptic systems can integrate diagnostic tools, enabling students to learn not just procedural skills but also critical thinking and decision‐making related to diagnosing dental issues. (Huang et al., 2023)	**Cost and resource allocation**: One of the significant barriers to widespread adoption of VR‐haptic technology is the financial investment required for equipment and training. Many institutions face challenges in allocating funds and space for these advanced systems, which can limit their implementation. (Perry et al., 2017; Al‐Saud, 2021) **Under‐researched integration**: While there is enthusiasm for VR‐haptic technology, the combination of these systems with traditional teaching methods remains under‐researched. There is a need for more comprehensive studies to fully understand how these technologies can complement existing educational frameworks effectively. (Hsu and Chang, 2023) **Limited availability**: Despite the benefits, fewer than 200 dental institutions worldwide have adopted VR‐haptic equipment due to various logistical challenges, including the need for trained personnel and ongoing maintenance of the technology. (Serrano et al., 2023) **Skepticism among educators**: Some educators express skepticism about the effectiveness of VR‐haptic training compared to traditional methods, questioning whether it can fully replicate the complexities of real‐world dental procedures. (Patil et al., 2023)

Modern dental education needs to adapt to the new and different opportunities and challenges presented by digital dental education methods and the “digital learners” of today's increasingly digitalized world. The aim of this report is to present prior and current research in the role of VR‐haptics in dental education, identify and consolidate the knowledge that dental health educators need to effectively implement its use in the curricula, and introduce representative VR‐haptics‐supported educational programs and events.

## 2 METHODS

A literature search was performed using PubMed to identify papers that have evaluated the role that haptics‐reinforced virtual reality‐based devices play in learning, practicing, and teaching in dental education. Combinations of the following search terms and subheadings were considered appropriate for this investigation: dentistry, education, haptic, teaching, training, and virtual reality. The publications chosen were restricted to those published between 2010 and 2024 that described dental education or training within the medical and dental sciences. Out of the 667 identified articles, 105 (16%) were advanced to screening based on title/abstract, and 42 (6%) were eligible for full‐text reading and the creation of this narrative report. The searching strategy to narrow down the list of candidate articles included keywords and phrases such as “VR‐haptic device,” “cariology,” “prosthodontics,” and “oral surgery.”

The selected articles focused on evaluating the educational effectiveness of haptics‐reinforced virtual reality‐based training for dentistry or stomatology students/residents. These criteria ensured that the studies specifically targeted the use and implementation of VR technology in the dental education field, providing valuable insights into its impact on learning outcomes in this specialized area of study. Only papers published in English were considered, and articles that were not peer‐reviewed were excluded.

The two‐stage screening process used by our independent reviewers involved an initial assessment of papers based on titles and abstracts against specific criteria. This method helped quickly filter out irrelevant studies, and allowed us to focus on those that met the criteria for further evaluation. By streamlining the reviewing process in this way, reviewers could efficiently identify potentially relevant research and save time in the overall screening process. After passing the initial screening, papers underwent a full‐text assessment in the second stage. This in‐depth evaluation ensured that only the most relevant and high‐quality papers progressed further in the review process. Moreover, manually analyzing the reference lists of selected papers helped uncover additional relevant manuscripts that might otherwise have been overlooked in the electronic database search or other grey literature sources. This meticulous approach ensured a comprehensive review of the existing literature, and helped to fill in any potential gaps in the research findings.

In addition to the literary overview, we have attached some of the initial findings from our ongoing educator survey on the use of VR‐haptics in dental education (results are as of the August 9, 2024). An electronic survey was distributed to all members of the VR‐Haptic Thinkers Consortium and an additional 910 oral healthcare educators from around the globe. The aim was to gather insights into their perceptions on VR‐haptic training across various preclinical and clinical dental courses. The voluntary survey included a variety of questions designed to assess matters such as when VR‐haptic technology is implemented during dental education and on what courses, as well as the various curricular challenges encountered. This global study was classified as a non‐human subject program evaluation, and it adhered to the ethical guidelines set forth by both the European and Finnish National Board on Research Integrity, with ethical approval granted by the University of Eastern Finland Committee on Research Ethics under reference No. 13/2023. The data collected from these anonymous surveys underwent blind analysis following collection. In this additional way, we wish to present the current challenges in the use of VR‐haptics from the perspective of experienced educators all around the world. We also list some of the suggested additions and improvements that the educators are currently hoping for.

## 3 RESULTS AND DISCUSSION

### 3.1 A new era

The dental educational sector is poised for digital disruption, and there has been a drastic increase in the applicational scope of digital solutions in preclinical and clinical education support around the globe. The field of VR‐haptic simulation has seen rapid growth since 2009 when the Simodont VR‐Haptic Dental Trainer was first introduced.^22^ Along with the rise in their utilization, VR‐haptic training tools are becoming increasingly more realistic and sophisticated. Their visual fidelity and user experience have significantly improved compared to what they were just 10 or 5 years ago, and as a consequence, an increase in the use of practice modules involving VR‐haptics is likely to occur in the early dentistry curriculum.^23^, ^24^ Haptic technology is especially appropriate during early dental manual skills training as VR‐haptic training has been shown to significantly increase the level of new students’ basic technical skills and confidence, even when only relatively simple models are used for practicing dental procedures. Despite this, there is currently no consensus about the best methods and practices in using VR‐haptics in the various dental curricula.^25^, ^26^, ^27^, ^28^, ^29^, ^30^, ^31^, ^32^

### 3.2 Benefits of VR‐haptics

VR‐haptics offers unique benefits and sustainable learning experiences and outcomes.^33^, ^34^ The mission of VR‐haptic dental training aligns with fostering a university training environment where “the learner is the protagonist.”^33^ For example, it can enable more cost‐effective dental education by reducing the amount of direct contact hours required from the educators while offering a more relaxed, independent training environment for the students within various dental disciplines.^35^ Virtual simulation allows students to work in a less stressful environment, as they can try the practices again and again unlimited times until they achieve their goal without the risk of irreversible damage to simulated materials or human teeth while using a phantom head or, even worse, performing on a real patient.^36^ In this respect, it is acknowledged that before starting patient treatment in clinical settings, dental students must demonstrate advanced psychomotor skills, which could be acquired via VR‐haptic‐based training. Typically, students perceive VR‐haptic simulation training as a transitional “bridge” connecting preclinical instruction and clinical practice.

Certain VR‐haptic dental simulators offer advantages such as ensuring proper ergonomic posture by requiring students to look through a projector window screen, and fostering a sense of competition by establishing targets for task completion at specified difficulty levels within a constrained timeframe. The ability of haptic trainers to mandate indirect vision in selected exercises has no equal analog in the physical world. Thus, this allows training students to have appropriate body awareness and avoid potential chronic injuries from abnormal neck and back posture while performing dental procedures.

The real‐time individualized feedback helps improve students’ confidence in their ability while pinpointing the areas in their skills that still require more attention. In traditional settings, students who are in need of extra training in manual dexterity skills often do not realize it themselves. With the help of VR‐haptic simulation, educators can offer them standardized as well as additional or customized training to develop their hand skills to reach the average expected dexterity level. It has also been shown that virtual simulation exercises enable skill transfer to real‐world settings. An important byproduct of utilizing VR‐haptics is sustainability, as the need for single‐use materials and active water supply and waste management is significantly reduced.

More importantly, many dental schools around the world find it more and more difficult to get individuals to volunteer as patients for undergraduate training. The haptic system will, if not give a fully clinical experience, at least mimic the clinical setting as close as is possible today. Expanding the databases of VR‐haptic systems with real clinical cases and synchronizing VR‐haptic training with phantom head‐based simulations could represent a very promising educational path; it is worth reiterating that VR‐haptics provides the opportunity to simulate real clinical scenarios before experiencing them with real patients. Consequently, the consolidation of competency can be directed within a simulated environment, ensuring that patient care is administered by clinicians with validated skills.^14^

Recently, it has been demonstrated that virtual dental simulation and haptics benefits extend beyond developing practical skills to improving didactic knowledge and students’ confidence levels.^35^ Applications of virtual dental simulation in dental education can span undergraduate dental programs to advanced dental implant training, endodontic microsurgery, and the planning of craniofacial and orthognathic surgery.^37^, ^38^, ^39^ In addition to the numerous applications listed above, we propose that virtual dental simulators also could be used as an adjunct tool on an ad hoc basis for senior students that are struggling with certain aspects of clinical practice.^40^, ^41^

### 3.3 Limitations to current VR‐haptics

The total number of dental schools worldwide is challenging to pinpoint with absolute accuracy due to variations in counting methods and updates in the establishment of new institutions. However, it is estimated that there are over 1000 dental schools globally. Despite the unique and attractive benefits of VR‐haptic trainers, during the past few years they have so far been installed in less than 15%–20% of the dental schools around the world.^31^ There are some obvious reasons for this: first, financial constraints: dental institutions have to allocate initial investment funds and faculty “buy‐in” time to facilitate the integration of digital dental simulators in their preclinical setups. Second, the time commitment: expert dentists are needed to design and create new evidence‐based curricula that utilize the VR‐haptic trainers. Third, space requirements: suitable facilities must be found for setting up training laboratories and the associated resources. These three reasons are interrelated in that faculty time impacts finances and space is costly. Additionally, while VR‐haptic technology holds considerable potential to improve dental educational experiences by fostering greater engagement, facilitating practical skill development, and allowing personalized learning, the absence of cross‐border comprehensive evidence‐based validation testing raises critical questions regarding its overall effectiveness. To fully capitalize on the benefits of these new technologies in educational settings and to address skepticism among educators, rigorous global research and the establishment of standardized practices are required. Such an approach will be pivotal in maximizing the impact of VR‐haptics on dental education.

However, it is also important to highlight that there are still some limitations to virtual dental simulation, such as the finger rest positioning, as well as the absence of specific training for light reflection and retraction of adjacent soft tissues using the students’ non‐dominant hand.^42^ Adverse physical (cybersickness/nausea) and mental (discomfort, fear) reactions are also possible, though unlikely.^43^ Furthermore, the applications of virtual dental simulation in, for example, endodontic training are still limited and restoring teeth with crowns and fillings is currently not supported either.^44^ Although there are many VR‐haptic simulators in the market, the three most commonly used ones, Simodont Dental Trainer, DENTE Simulator, and Virteasy Dental Simulator, have been discussed in Table 2 to showcase some of the features that are and are not available (adapted and modified from Imran et al., 2021).^23^, ^45^

**TABLE 2:** Comparison of the key features of different haptics‐enhanced virtual reality simulators.

Feature	Simodont dental trainer	SIMtoCAREDENTE simulator	Virteasy dental simulator
Type of device	VR‐haptic	MR‐haptic	VR‐haptic
Allows ergonomic posture	Yes	Yes	No
Offers instant feedback	Yes	Yes	Yes
Allows educator to examine the exercises	Yes	Yes	Yes
Allows Wi‐Fi connection	No	Optional	Optional
Allows the instructor to view the simulators live at the same time and record all exercises, their evaluation, and subsequent comments	Yes	Yes	Yes
Can be used by both right‐ and left‐handed users	Yes	Yes	Yes
Allows off‐campus use	No	Possible (with an additional server)	Yes
The 3D images are realistic, but the texture of the healthy, decayed, and/or restored tooth still needs improvement	Yes	Yes	Yes
Device offers anesthesia, periodontics, and implantology exercises (in addition to dexterity exercises, restorative dentistry, pediatric dentistry, prosthetics, and endodontics)^a^	No	All	Periodontics, implants, and anesthesia
Simulation exam capability	Yes	Yes	Yes
Animated teeth	Yes	Yes (combination of virtual and real teeth)	Yes
Possibility of .dcm and/or .stl file upload for patient‐specific scan virtualization and training	Yes	Yes	Yes
App for students	No	Yes	No

*Note*: Adapted and modified from Imran et al. (2021).

^a^ As of May 2024.

### 3.4 Summary of the advantages and limitations of VR‐haptics

Comparing VR‐haptic training to traditional dental training methods reveals several advantages and limitations to each approach, particularly in the context of dental education and other practical skill training (Table 3).

**TABLE 3:** Advantages and limitations of VR‐haptic training.

***Advantages of VR‐haptic training***	
**Enhanced realism and immersion**: VR‐haptic training provides a more immersive experience by simulating real‐world scenarios, while allowing learners to engage with content in a way that traditional methods either cannot provide or cannot do so as readily. The integration of haptic feedback allows users to feel and manipulate virtual objects, enhancing the realism of the training experience. **Improved skills acquisition**: Studies indicate that VR‐haptic training can significantly enhance practical skills and theoretical knowledge. For instance, research has shown that students using VR‐haptic simulators perform better in practical examinations compared to those undergoing traditional training methods. The tactile feedback helps in developing muscle memory, which is crucial for tasks requiring fine motor skills, such as dental procedures. (Koolivand et al., 2024) **Immediate and objective feedback**: VR‐haptic systems can provide instant feedback on performance, allowing learners to correct their mistakes in real time. This immediate evaluation is often lacking in traditional training environments, where feedback may be delayed or less specific. (Felszeghy et al., 2023) **Safety and cost‐effectiveness**: VR‐haptic training eliminates many of the risks associated with real‐world practice, particularly in high‐stakes environments like healthcare. It also reduces costs related to materials and equipment needed for traditional training. (Koolivand et al., 2024) **Increased confidence**: Trainees often report feeling more confident in their abilities after VR‐haptic training. For example, a study found that VR‐haptic learners were 275% more confident in their skills compared to those trained traditionally. (Felszeghy et al., 2023)	
***Limitations of VR‐haptic training***	
**Accessibility and cost of technology**: While VR‐haptic training offers many benefits, the initial investment in the technology can be high. Not all institutions may have the resources to implement VR‐haptic systems, which can limit access for some students. (Leung et al., 2021) **Learning curve for technology**: Some educators and learners may find it challenging to adapt to VR‐haptic technology, especially those who are less familiar with digital tools. This can create a barrier to effective learning if not addressed properly. (Hsu and Chang, 2023)	

### 3.5 Implications for curriculum development

There appears to be a sense of uncertainty surrounding the integration of VR‐haptic devices in dental education, particularly with the influx of new companies entering the market. VR‐haptic dental trainers must meet the expectations set on new teaching methods. Consequently, a need for thorough evaluation and additional research has emerged to better understand the effects of the implementation of these new educational tools across undergraduate and postdoctoral curricula.^35^ It has been reported that the integration of virtual dental simulation into dental curricula should be planned in a strategic and methodological way in order to bridge the gap between preclinical and clinical training, allowing for constructive alignment between the different components of the dental programs.^25^, ^26^ In short, sufficient time and resources must be invested.

Effective VR‐haptics‐reinforced dental education requires thorough curriculum redesign. The integration should not be viewed as a simple technical conversion from phantom head‐based simulation to VR‐haptic simulation. Dental faculty should evaluate whether to incorporate VR‐haptic components directly into all courses with traditional phantom head simulation, limit them to specific disciplines, or even mostly just offer them as alternatives to students who require additional practice or who might otherwise miss simulation laboratory training due to unavoidable causes like illness.

Although VR‐haptic education is likely to become more and more important for practicing different case‐specific tooth preparations, phantom head simulation laboratory sessions and clinical training with patients should still remain an integral part of dental education. VR‐haptic technology should therefore not be seen as a replacement for but an integral adjunct of conventional training using phantom head‐based and clinical teaching.^27^ One incentive for the faculty to use haptic training as part of their student development is the opportunity for immediate, consistent, and objective assessment. This is something students immediately appreciate, and it creates a market pull dynamic.

When designing a new dental curriculum, regardless of the dental discipline, content delivery needs to be tightly linked with the chosen conventional tools. Careful alignment between learning tasks, outcomes, and performance measures must be maintained.^46^, ^47^ Our results demonstrate a lack of consistency between the studies conducted in the field of virtual dental simulation due the utilization of different technologies and educational protocols. Our findings indicate the need for standardization between studies as well as the need for cross‐institutional studies that are deemed necessary to develop international guidelines in this field. Finally, it is important to spread the message about virtual dental simulation amongst dental educators who are still resistant or hesitant to implement this technology due to the fear of the unknown and the challenges they face with change management.

### 3.6 VR‐haptic educator survey

#### 3.6.1 Information on survey entries

The future of VR‐haptics‐supported education holds great promise as the technology continues to advance and becomes more accessible, allowing its applications in education and training to expand significantly. There is great potential for this technology to revolutionize how students learn and professionals train in a wide range of fields.

**FIGURE 1:**
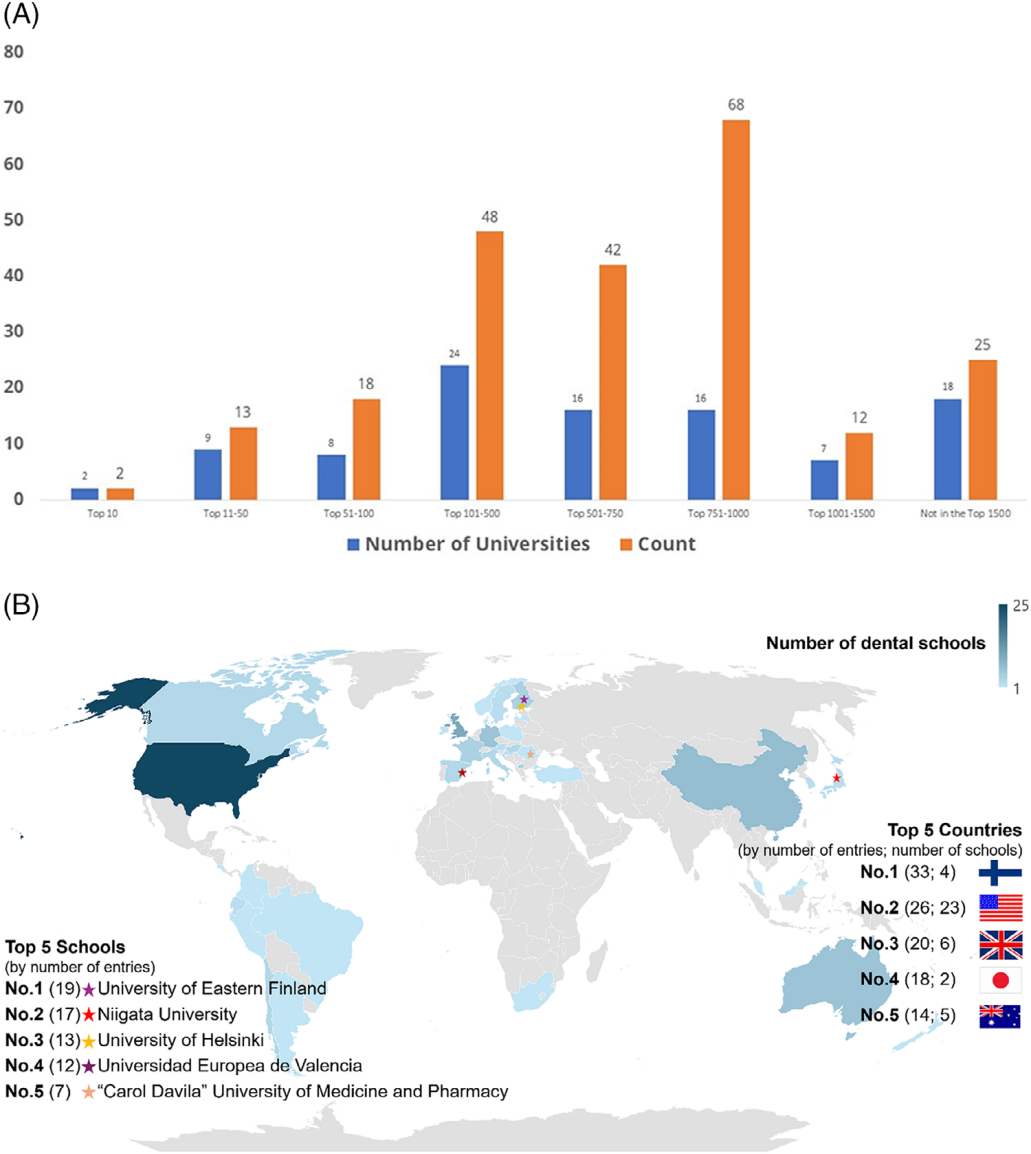
Graphical representations of the educator survey entries and their sources (as of early August 2024). The bar chart shows the number of survey entries (*n* = 228) from the QS World University Ranking 1: 1500 universities. Respondents included 140 people who had DDS degrees, of whom 90 also reported having a PhD. There also were 60 responses from PhDs without DDS degrees. The map chart provides information on where the replies came from geographically, as well as the top five countries and schools from which replies have been received.

Through our ongoing educator survey, we have received replies from 228 dental educators and researchers by early August 2024. The educator experiences gathered through the VR‐Haptic Thinkers’ survey come from many of the top universities in the world (Figure 1). This helps lend credence to the replies as: (i) many employers often perceive degrees from these institutions as indicators of quality education and preparedness; (ii) studying at leading universities often exposes students to diverse cultural and academic environments; and (iii) top universities are often at the forefront of research and innovation.

#### 3.6.2 VR‐haptics in dental education: Reported challenges and improvement suggestions

Roughly 25% of the respondents have provided freehand comments on challenges that hinder the implementation and use of VR‐haptic devices during preclinical and clinical phases. These comments offer valuable insight into what needs to be improved to enable VR‐haptics’ effective use in education, guiding future research and innovation in this field. Table 4 shows the top five most common comments on hardware‐ and software‐related challenges. In addition, 56% of the respondents gave direct suggestions for what needs to be added to VR‐haptic devices. The top five themes and examples of the suggested additions are shown in Table 5.

**TABLE 4:** Educator survey: Top five reported VR‐haptic device‐based challenges (as of early August 2024).

**Preclinical phase challenges**	
Devices missing clinical metrics and/or easily accessible real‐time performance analysis options Lack of high‐ and low‐speed options for instrumentation No voice feedback and/or voice controls Better (possibly AI‐powered) personalization options are needed Insufficient realism/precision, the technology is not “there” yet	
**Clinical phase challenges**	
Devices missing clinical metrics and/or easily accessible real‐time performance analysis options Insufficient realism/precision, the technology is not “there” yet Lack of high‐ and low‐speed options for instrumentation Transfer of real‐life cases to devices and/or their utilization therein is too difficult No voice feedback and/or voice controls	

*Note*: Preclinical phase commenters (*n* = 58) included 53 people who had DDS degrees, of whom 37 also reported having a PhD. Clinical phase commenters (*n* = 57) included 51 people who had DDS degrees, of whom 36 also reported having a PhD.

**TABLE 5:** Educator survey: Top five reported suggestions for what should be added to VR‐haptic devices (as of early August 2024).

Addition suggestion themes	Examples
Additional training scenarios	Extraction, implantology, oral surgery, injections, anesthesia, periodontics, dental fillings, identification of caries lesions, flap design, tooth transplantation, root tip resection, calculus removal, etc.
Software improvements	More personalization options, voice feedback, voice controls, improved simulation of tissues, addition of surrounding structures, improved assessment options, easier upload, and use of patient scans, etc.
AI‐based improvements	Personalized training for students based on their strengths and weaknesses, automated performance assessment on both individual and group levels, automated tutoring, etc.
Equipment improvements	Portable devices, high‐ and low‐speed options for instrumentation, additional and more varied instrumentation for different training scenarios and treatments (lights, different grit sizes, etc.), cameras for tracking user posture, VR‐headset use availability (in addition to 3D monitors), etc.
Gamification	Allow users to “play” against each other for high scores (speed, precision, etc.) both locally and globally over the internet, add cooperation possibility for simultaneous work on the same case, etc.

*Note*: Commenters (*n* = 127) included 99 people who had DDS degrees, of whom 64 also reported having a PhD.

The most suggested enhancements for VR‐haptic devices include additional training scenarios, software improvements, and AI‐based personalized training enhancements. These upgrades would provide users with a more versatile and engaging training experience, while tailoring their training to fit their individual needs. By incorporating these improvements, VR‐haptic devices could offer a more widely applicable, effective, and personalized training platform for a range of dental applications.

### 3.7 Future vision

Substantial diversity exists in the purpose, structure, processes, and content of VR‐haptics‐reinforced conventional dental education across the world. Only through collaboration can these aspects be synchronized, and the challenges posed by VR‐haptics in dental education, research, and practice be mitigated. The first conference on the use of VR‐haptics in dental education, the VR‐Haptic Thinkers Meetup, was held in November 2023. The success of the meetup and the new connections enabled the formation of an international VR‐Haptic Thinkers Consortium in February 2024. With members from the American Dental Education Association (ADEA), Association of Dental Education in Europe (ADEE), and other respected national and international associations of dental health educators and researchers, the focus was set on five major areas: performing cross‐border cutting‐edge educational research; providing support for developing VR‐haptic niches; promoting VR‐haptics‐supported curriculum development; disseminating data via free, hybrid meetups; and deepening the discussion between the members of the academics and industry.

To effectively disseminate and integrate VR‐haptic technology in dental education, educators, institutions, and researchers should undertake several strategic steps (Table 6). These actions will optimize the use of VR‐haptics, enhance learning outcomes, facilitate broader acceptance and utilization of the technology within dental curricula, and expand the existing evidence‐based knowledge pool globally.

**TABLE 6:** Steps for integration and optimization of the use of VR‐haptics in the dental curricula.

***Curricular development and integration:***
**Curriculum design**: Collaborate to design curricula that incorporate VR‐haptic training alongside traditional methods, ensuring a balanced approach that leverages the strengths of both. This includes defining when and how VR‐haptic training should be introduced throughout the dental education program. **Pilot programs**: Initiate pilot programs in select courses to evaluate the effectiveness of VR‐haptic training and gather data on student performance and satisfaction. This will help in refining the institutional integration process based on empirical evidence.
***Faculty training and support:***
**Professional development**: Provide regular training sessions for faculty members on the use of VR‐haptic technology, emphasizing its benefits and practical applications in dental education. This will help educators feel more comfortable and proficient in using the technology. **Peer collaboration**: Encourage collaboration among faculty to share best practices, challenges, and successes in implementing VR‐haptic training. Establishing a VR‐haptic community spirit can foster innovation and continuous improvement.
***Research and evidence generation:***
**Conduct studies**: Engage in research to assess the impact of VR‐haptic training on student learning outcomes, skill acquisition, and confidence levels. This research should also explore the comparative effectiveness of VR‐haptics versus traditional training methods where possible. **Publish your findings**: Share research findings through academic journals and conferences to contribute to the body of knowledge surrounding VR‐haptic technology in dental education. Disseminating results will help encourage adoption by and validate the approaches of other institutions.
***Resource allocation and infrastructure development:***
**Investment in technology**: Advocate for institutional investment in VR‐haptic equipment and infrastructure to ensure adequate access for students. This may involve seeking external funding or partnerships with technology companies.
***Student engagement and feedback:***
**Incorporate student input**: Actively involve students in the development and implementation process by soliciting their feedback on the VR‐haptic training experience. Understanding their perspectives can lead to improvements in and increased acceptance of VR‐haptic technology. **Promote awareness**: Educate students about the benefits of VR‐haptic training and how it complements traditional methods. Highlight success stories and improvements in skills and confidence resulting from its use.
***Networking and c ollaboration :***
**Establish networks**: Join or create networks of educators, researchers, and dental professionals interested in VR‐haptics. This facilitates knowledge sharing, collaborative research, and collective advocacy for VR‐haptic technology. **International collaboration**: Engage in international discussions and meetups focused on VR‐haptic technology in dental education to share insights, challenges, and innovations across borders. **Communicate with the manufacturers**: Do not be afraid to contact and discuss issues and desired improvements in your VR‐haptic devices with the manufacturers. Your feedback is essential in product development. If nothing else, discuss the challenges you have encountered within your peer network and collaborators so that a better understanding of what might be missing can be formed.

## 4 CONCLUSION

This report highlights the transformative potential of haptics‐reinforced virtual reality training in dental education. VR‐based training offers standardized learning opportunities, fostering high engagement and knowledge transfer to clinical practice. It addresses educator shortages and enables flexible, individualized learning, with promising applications for future dental education. VR‐haptic scenarios enhance student learning experiences, particularly in times of restricted face‐to‐face training like the COVID‐19 pandemic. However, several questions remain unanswered. Effective supervision and assessment are crucial, along with understanding optimal instruction and feedback characteristics for skill transfer. Cost‐effectiveness comparisons and studies on decision‐making improvement are needed. Rigorous research standards and tracking performance are essential, along with integrating VR‐haptic education into workforce training and maintenance. Ongoing evaluation and dissemination of educational outcomes are vital, as is exploring broader applications beyond current dental competencies.

## CONFLICT OF INTEREST STATEMENT

The authors declare that they have no known competing financial interests or personal relationships that influenced the work reported in this paper.
